# *Brevundimonas* and *Serratia* as host systems for assessing associated environmental viromes and phage diversity by complementary approaches

**DOI:** 10.3389/fmicb.2023.1095850

**Published:** 2023-03-21

**Authors:** Ines Friedrich, Hannes Neubauer, Alisa Kuritsyn, Bernhard Bodenberger, Faina Tskhay, Sara Hartmann, Anja Poehlein, Mechthild Bömeke, Michael Hoppert, Dominik Schneider, Robert Hertel, Rolf Daniel

**Affiliations:** ^1^Genomic and Applied Microbiology, Göttingen Genomics Laboratory, Institute of Microbiology and Genetics, Georg-August University of Göttingen, Göttingen, Germany; ^2^General Microbiology, Institute of Microbiology and Genetics, Georg-August University of Göttingen, Göttingen, Germany; ^3^FG Synthetic Microbiology, Institute of Biotechnology, BTU Cottbus-Senftenberg, Senftenberg, Germany

**Keywords:** *Brevundimonas*, *Serratia*, dsDNA phages, host-associated viromes, host-associated dsDNA virome, broad host-range phage

## Abstract

Focusing on visible plaques for phage isolation leaves the question if we miss the diversity of non-plaque forming phages. We addressed this question through direct plaque-based isolation by employing the new hosts *Brevundimonas pondensis* LVF1 and *Serratia marcescens* LVF3 dsDNA, ssDNA, dsRNA, and ssRNA host-associated metavirome analysis. Of the 25 distinctive dsDNA phage isolates, 14 were associated with *Brevundimonas* and 11 with *Serratia*. TEM analysis revealed that 6 were myoviruses, 18 siphoviruses and 1 podovirus, while phages infecting *Brevundimonas* belonged all to siphoviruses. The associated viromes suggested a higher phage diversity in summer than in winter, and dsDNA phages were the dominant group. Isolation of vB_SmaP-Kaonashi was possible after investigating the viromes associated with *Serratia*, demonstrating the great potential of accompanying host-associated metavirome analysis. The ssDNA virome analysis showed that the *B. pondensis* LVF1 host is associated with *Microviridae* and *Inoviridae* phages, although none of them were isolated. The results demonstrated that the classical isolation technique is not exhausted, leading to the isolation of new dsDNA phages. It can be further improved by combination with metavirome techniques, which revealed further diversity.

## Introduction

Bacteriophages or phages are bacterial viruses that infect and replicate in bacterial cells and belong to the most diverse entities of the planet (Casas and Rohwer, 2007; Dion et al., 2020). With an estimated number of 10^31^ virions on earth, phages outnumber bacterial cells in various environments by approximately ten-fold (Dion et al., 2020). The highest phage densities were observed in wastewater treatment plants (WWTP) (Wu and Liu, 2009).

As intracellular parasites, phages rely on their host metabolism for replication. The host range is phage-specific and may include single or multiple bacterial species (Garmaeva et al., 2019). They either reduce the population through direct replication (lytic route) (Carding et al., 2017) or enter a long-term relationship with the host by integrating into the host genome as a prophage (lysogenic route) (Principi et al., 2019). Prophages provide additional genetic information and can supply the host with extra properties resulting in a competitive advantage.

Today bacteriophages are classified based on their genome sequence and organization (Dion et al., 2020). The resulting groups usually correlate with viral morphology. Some have a head-tail morphology (*Caudoviricetes*), others are filamentous (*Inoviridae*), pleomorphic (*Plasmaviridae*) or polyhedral (*Microviridae*, *Corticoviridae*, *Tectiviridae*, *Cystoviridae*, and *Leviviricetes*). In addition to the viral capsid, internal or external lipid membranes may also exist. Unlike other phages, pleomorphic phages do not have capsids and form a proteinaceous lipid vesicle. The phage genetic material comprises RNA or DNA, varying from single- to double-stranded and from linear to circular while no circular RNA phages have been reported so far (Dion et al., 2020). Most of the characterized phages isolated to date are tailed and use dsDNA as genomic material (Dion et al., 2020; Zrelovs et al., 2020). Furthermore, some groups are particularly prominent regarding the virus type and the genome size (Zrelovs et al., 2020).

To explore virus types and genome sizes, we used *Brevundimonas pondensis* LVF1 (Friedrich et al., 2021b) and *Serratia marcescens* LVF3 (Friedrich et al., 2021a) as host systems. *B. pondensis* is an oligotrophic bacterium and belongs to the family *Caulobacteraceae*. This strain has a single flagellum, is Gram negative, aerobic and grows best at 30°C. *Serratia marcescens* LVF3 belongs to the family *Yersiniaceae*. It is Gram negative, possesses a flagellum, and is a copiotrophic organism. The optimal growth temperature is also 30°C. Both host systems are excellent for studying viral diversity, as both have yielded a variety of different plaques by plaque assay in preliminary experiments. We isolated individual phages and investigated the viral community associated with the two hosts by viral metagenome analysis. Thereby, we assessed not just dsDNA material but also ssDNA, dsRNA, and ssRNA viromes. This was done using specific nucleases receiving the purified form of aforementioned viromes. We used sewage samples from a WWTP from two different seasons (winter and summer) as source material. Isolates were characterized by morphology, genome sequence, and alignment to the metavirome-derived sequencing data to explore the hidden potential of discovering new phages.

## Materials and methods

### Phage isolation and host-based phage enrichment

1 L primary treatment sewage from the municipal WWTP in Göttingen, Germany, collected in January 2019 (used for infection of *Brevundimonas pondensis*), July 2019 (used for infection of *Brevundimonas pondensis* and *Serratia marcescens*), and January 2020 (used for infection of *Serratia marcescens*), served as environmental phage sources. Samples were centrifuged at 6,000 × *g* for 15 min. The supernatant containing phages was sterile-filtered employing a 0.45 μm non-pyrogenic PES-membrane (Sarstedt AG & Co. KG, Nümbrecht, Germany). Phages were precipitated by adding polyethylene glycol (PEG) in a final concentration of 10% (w/v) and 0.5 M NaCl. After incubation at 4°C for 16 h, phages were harvested by centrifugation at 10,020 x *g* and 4°C for 1 h. The supernatant was discarded and phage pellets were suspended in 25 mL PYE (0.2% peptone, 0.1% yeast extract, 0.02% MgSO_4_ × 7 H_2_O) for phages associated with *B. pondensis* LVF1^T^ and in TSB-10 (1.7% peptone from casein, 0.3% peptone from soybean, 0.25% K_2_HPO_4_, 1% NaCl, 0.25% glucose monohydrate) for phages associated with *S. marcescens* LVF3^R^ (Friedrich et al., 2021b).

A total of 1 mL of the prepared suspension was used for the infections of *B. pondensis* and *S. marcescens*. Phages were isolated *via* agar overlay plaque assay as described elsewhere (Kropinski et al., 2009) using host-specific culture media for the basal agar (1.5% agarose) and 2.5 mL overlay (0.4% agarose). Infected overlay plates were incubated overnight at 30°C. Morphologically distinct plaques representing individual phage isolates were picked with a sterile toothpick, and each was transferred to 500 μL sterile culture medium. Further phage strain purification was performed *via* three subsequent reinfections, resulting in pure cultures.

To harvest the virome associated with the host, the initial overlay was washed with 4 mL of the respective medium, also allowing to harvest phages which might not be able to form plaques under the given conditions. The phage suspensions were processed as described below. Salt Active Nuclease (SERVA, Heidelberg, Germany) was added to the phage suspensions (20 U/mL) prior to precipitation to digest non-particle protected host-associated nucleic acids.

### Purification of viral nucleic acids and preparation of viral dsDNA, ssDNA, dsRNA, and ssRNA

All kits and enzymes were used as recommended by the manufacturer if not otherwise stated. The MasterPure™ Complete DNA and RNA Purification kit (Lucigen, Middleton, WI, United States) was used with modifications to extract total viral nucleic acids. Due to the high protein content, we increased the amount of Proteinase K (20 mg/mL) to 5 μL in 300 μL of 2X T and C Lysis Solution, which was applied to 300 μL of phage suspension. We obtained pure viral genomic DNA by applying RNase A (DNase free) to the total nucleic acid preparation and DNase I (RNase free) for pure viral RNA. Before sequencing of dsDNA phages, the extracted DNA of putative phages was digested with the *EcoR*I restriction endonuclease (Fisher Scientific GmbH, Schwerte, Germany) by determining a unique restriction digestion pattern of phages and therefore eliminating duplicates.

To receive ssDNA, dsDNA was removed *via* dsDNA-specific dsDNase (Thermo Fisher Scientific, Waltham, MA, United States). Viral ssDNA was *in vitro* transformed to dsDNA using Klenow fragment (Thermo Fisher Scientific, Waltham, MA, United States) and random hexamer primers (Thermo Fisher Scientific, Waltham, MA, United States). S1 nuclease (Thermo Fisher Scientific, Waltham, Ma, United States) was applied to the total nucleic acids to remove single-stranded molecules for dsDNA and dsRNA purification. RNase III (Thermo Fisher Scientific, Waltham, MA, United States) was used to remove dsRNA for ssRNA purification.

### Phage genome and host-associated virome sequencing and sequence read processing

Phage genomes were sequenced with an Illumina MiSeq-system (2 × 300 bp) as described previously (Kohm et al., 2022). RNA samples were reverse transcribed to dsDNA *in vitro* and sequenced like dsDNA samples with an Illumina MiSeq-system (2 × 300 bp) as described previously (Kohm et al., 2022).

Potential host reads were removed by mapping to the host genome employing bowtie2 (Langmead and Salzberg, 2012). Unmapped pairs were quality-processed employing Trimmomatic v0.39 (Bolger et al., 2014) and paired reads joined with FLASH v1.2.11 (Magoč and Salzberg, 2011). The quality-processed reads served as input for the Unicycler v0.4.9 assembly pipeline in normal mode (Wick et al., 2017), which included Spades v3.13.0 (Bankevich et al., 2012), makeblastdb v2.11.0+ and tblastn v2.11.0+ (BLAST® Command Line Applications User Manual [Internet], 2019), bowtie2 v2.4.4 (Langmead and Salzberg, 2012), SAMtools v1.12 (Li et al., 2009), java v.11.0.13 (Arnold et al., 2005), and Pilon v1.23 (Walker et al., 2014). Assembly was quality-assessed using QualiMap v2.2.2 (Okonechnikov et al., 2016). Genomes of individual phage isolates were annotated with VIBRANT (Kieft et al., 2020) and InterProScan v5.55–88.0 (Zdobnov and Apweiler, 2001), and data were submitted to GenBank (Benson et al., 2017).

Raw reads from the ssDNA, dsRNA, and ssRNA viromes (Supplementary Table S1) were mapped to the assembled genome using bowtie2 v2.4.4 (Langmead and Salzberg, 2012) to remove putative dsDNA contamination. Unmapped reads were used for virome assembly with the Unicycler v0.4.9 assembly pipeline in normal mode (Wick et al., 2017). The resulting contigs were searched against BLAST nt database v2.12.0+ (accessed on July 14, 2022) (Altschul et al., 1990; Altschul, 1997; Camacho et al., 2009) to identify further contamination. Contigs derived from the ssDNA, dsRNA and ssRNA virome with significant similarities to prokaryotic sequences were considered contaminations and excluded from the analysis. Such contamination was only observed in samples from the summer season and only in the ssDNA virome of *B. pondensis* LVF1 and from both seasons in the RNA viromes of *S. marcescens* LVF3. The remaining contigs (larger than 1,000 bp) were mapped on all publicly available and our isolated phage genomes (Supplementary Table S2) using pyani ANIb method (Pritchard et al., 2016). Contigs which showed a match with at least 70% nucleotide-to-nucleotide sequence identity to known phages were not further analyzed. The same method was applied to investigate high sequence identity (>90%) of dsDNA phage contigs with ssDNA or ssRNA contigs. Contigs which showed a match in the ssDNA/ssRNA virome were not further investigated. Further, contigs which did not reach a coverage over 30 (output of QualiMap v2.2.2), were removed. All dsDNA and resulting ssDNA, dsRNA and ssRNA contigs as well phage genomes were annotated with VIBRANT (Kieft et al., 2020) and InterProScan v5.55–88.0 (Zdobnov and Apweiler, 2001). Viromes, which could not be annotated *via* VIBRANT, were annotated with Phage Commander (Lazeroff et al., 2021) including RAST v2.0 (Aziz et al., 2008), MetaGene (Noguchi et al., 2006), GeneMark v2.5 (Borodovsky and McIninch, 1993), GeneMark.hmm v3.25 (Besemer et al., 2001), GeneMark with Heuristics v3.25 (Zhu et al., 2010), GeneMarkS v4.28 (Besemer et al., 2001), GeneMark S2 (Lomsadze et al., 2018), Glimmer v3.02 (Delcher et al., 2007), and Prodigal v.2.6.3 (Hyatt et al., 2020), as well ARAGORN v1.2.41.c for identification of phage tRNAs (Laslett and Canback, 2004). Data of phage isolates and raw read sequences were submitted to GenBank (Benson et al., 2017).

### Taxonomic classification of *Brevundimonas*- and *Serratia*-associated phages

Taxonomic classification was performed using pyani v0.2.11 (Pritchard et al., 2016) with the ANIm option. Average nucleotide identity (ANI) values ≥95%, presented in white to red, indicate isolates of the same species. ANI values between ≤95 to 70%, presented in white to blue, indicate strains of the same genus (Parks et al., 2019).

**Figure 1:**
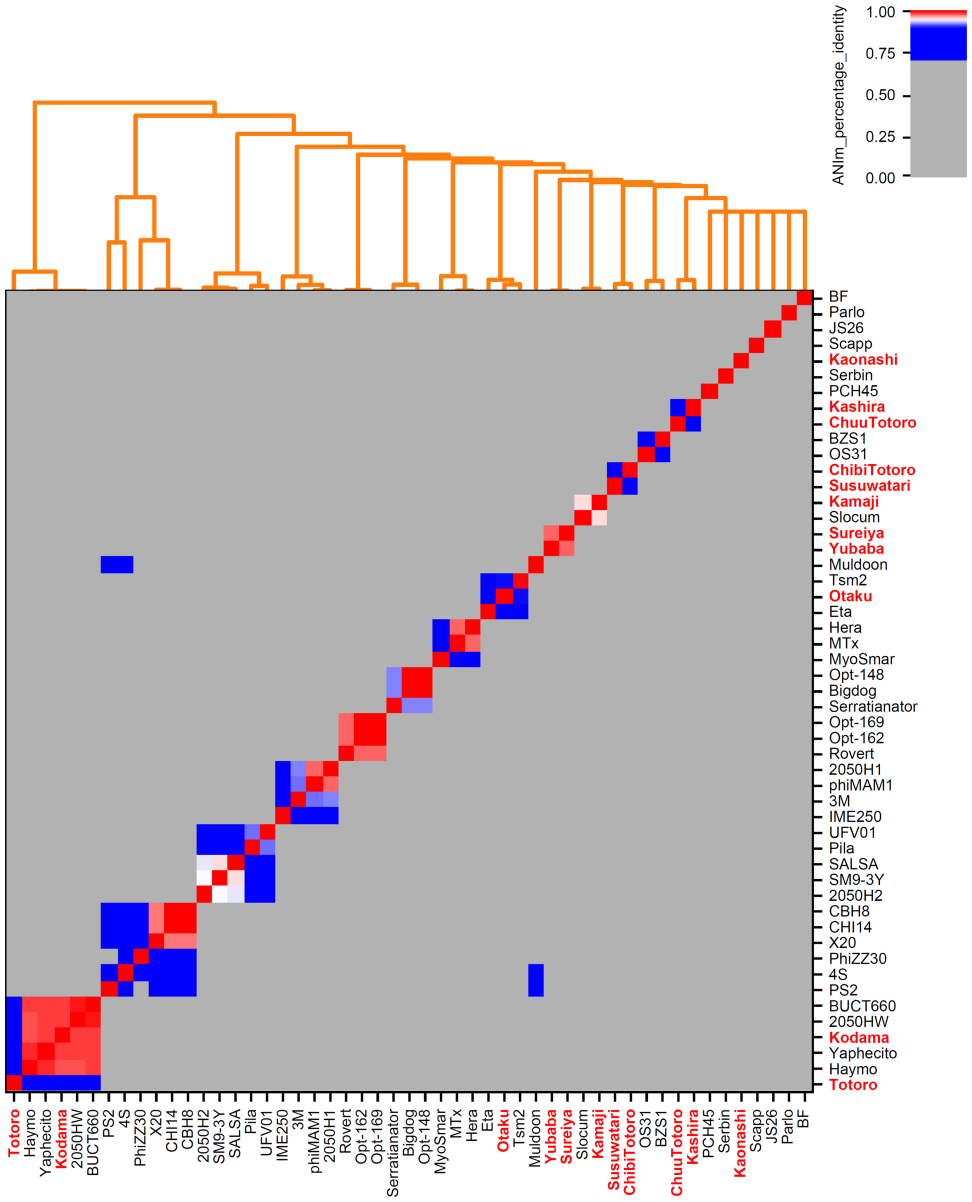
Genome-based phylogenetic analysis of *Serratia*-associated bacteriophages. All genomes from NCBI Virus (Brister et al., 2015) and our own isolates (marked in bold red) were examined. Calculations were done with pyani (Pritchard et al., 2016) using ANIm method with default parameters.

**Figure 2:**
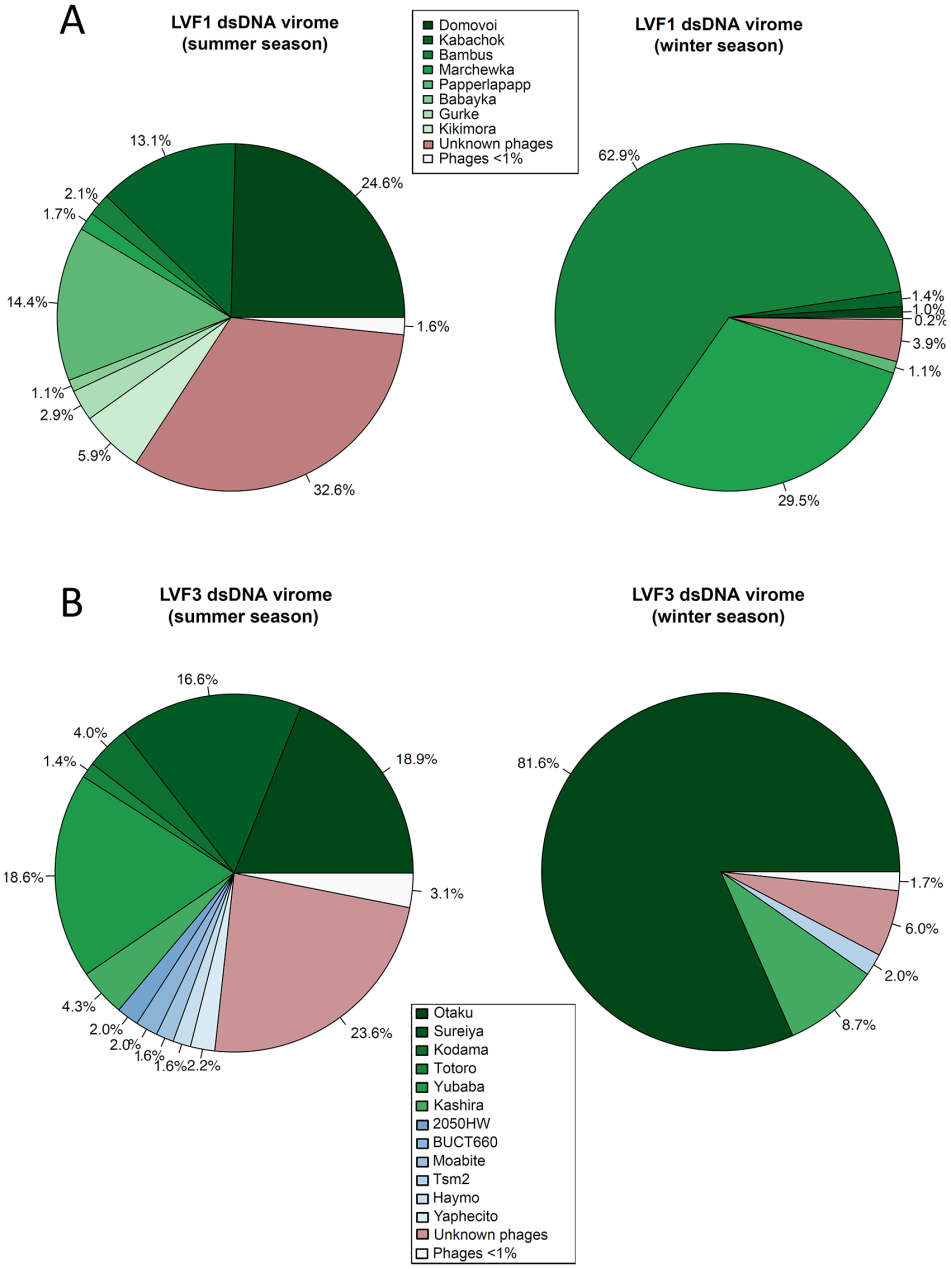
Mapping of viromes against all bacteriophage genomes available from NCBI as well our phage genomes from the isolates. **(A)** Pie chart of *B. pondensis* LVF1-associated dsDNA virome showing mapping against bacteriophage genomes associated with the family *Caulobacteraceae*. **(B)** Pie chart of *S. marcescens* LVF3-associated dsDNA virome depicting mapping against bacteriophage genomes associated with the genus *Serratia*. Number of mapped reads in relation to overall alignment is in percent. Visualized using RStudio (RStudio Team, 2020).

Bacteriophages associated with the family *Caulobacteraceae* (*Brevundimonas-*associated phages) and genus *Serratia* were downloaded from NCBI Virus (Brister et al., 2015) (accessed December 01, 2021). These included *Brevundimonas*- and *Caulobacter*-associated phages and *Serratia*-associated phages (Supplementary Table S2). An overview of all bacteriophages is provided in Supplementary Tables S3, S4 and Figures 1, 2.

### Morphology of phage isolates

Phage morphology was assessed by transmission electron microscopy (TEM). Data were imaged using the Digital Micrograph software (Gatan GmbH, Munich, Germany). The phage isolates were amplified and then, a negative staining technique was performed. For this purpose, a thin carbon film, evaporated by glow discharge onto freshly cleaved mica, was partly floated off on a drop of phage suspension. The mica was washed briefly with demineralized water and transferred to a thin copper-coated grid (PLANO GmbH, Marburg, Germany) and dried using a filter paper without touching the grid’s surface. The grid was stained using 50 μL of 2% uranyl acetate droplet with the carbon film facing downwards for 1 s. The grid was dried carefully and ready for the TEM imaging. Electron microscopy was performed with a Jeol 1011 transmission electron microscope (Jeol Ltd., Eching, Germany) equipped with a Gatan Orius SC1000 CCD camera (Gatan, Munich, Germany).

### Nomenclature of bacteriophage isolates

Isolates were named based on the informal guide by Adriaenssens and Brister (2017). Accordingly, vB stands for virus of bacteria, Bpo, and Sma for the host organism (*B. pondensis* and *S. marcescens*, respectively), M for the myovirus and S for siphovirus and P for podovirus, followed by an individual naming which does not follow any rules. Consequently, the full names of the viruses compose to, e.g., vB_SmaM-Otaku abbreviated Otaku.

## Results

### Phage isolation and characterization

*Brevundimonas pondensis* LVF1 and *S. marcescens* LVF3 served as hosts for plaque assay-based phage isolation (Supplementary Figure S1), which was performed with sewage samples obtained in winter 2019, summer 2020, and winter 2020. 25 (2019) and 50 (2020) individual plaques associated with *B. pondensis* LVF1 and 25 (2019) and 50 (2020) plaques associated with *S. marcescens* LVF3 were picked. Redundancies were eliminated by determining specific genomic restriction patterns of all isolates. This analysis also revealed that all genomes of isolates were comprised of dsDNA. Subsequently, 25 unique phages were obtained, 14 associated with *B. pondensis* LVF1 and 11 with *S. marcescens* LVF3 (Table 1).

**Table 1:** Morphological properties of all bacteriophage isolates associated with *B. pondensis* and *S. marcescens*.

Picture ID	Host	Morphotype	Name	Season	Phage size [nm]			
					Capsid width	Capsid length	Tail length	Total length
A	*B. pondensis* LVF1	Siphovirus	vB_BpoS-Papperlapapp	July 2019	53	363	400	763
B	*B. pondensis* LVF1	Siphovirus	vB_BpoS-Kabachok	January 2019	53	377	398	775
C	*B. pondensis* LVF1	Siphovirus	vB_BpoS-Domovoi	January 2019	55	381	381	762
D	*B. pondensis* LVF1	Siphovirus	vB_BpoS-Marchewka	January 2019	42	267	257	524
E	*B. pondensis* LVF1	Siphovirus	vB_BpoS-Bambus	January 2019	56	368	392	760
F	*B. pondensis* LVF1	Siphovirus	vB_BpoS-Gurke	July 2019	75	306	241	547
G	*B. pondensis* LVF1	Siphovirus	vB_BpoS-Kikimora	January 2019	92	278	317	595
H	*B. pondensis* LVF1	Siphovirus	vB_BpoS-Poludnitsa	July 2019	57	63	220	283
I	*B. pondensis* LVF1	Siphovirus	vB_BpoS-Leszy	January 2019	–	98	257	355
J	*B. pondensis* LVF1	Siphovirus	vB_BpoS-StAshley	January 2019	–	45	254	299
K	*B. pondensis* LVF1	Siphovirus	vB_BpoS-MaInes	January 2019	–	61	183	244
L	*B. pondensis* LVF1	Siphovirus	vB_BpoS-Strzyga	July 2019	–	60	155	215
M	*B. pondensis* LVF1	Siphovirus	vB_BpoS-Polewnik	July 2019	–	58	149	208
N	*B. pondensis* LVF1	Siphovirus	vB_BpoS-Babayka	July 2019	–	65	163	228
O	*S. marcescens* LVF3	Myovirus	vB_SmaM-Totoro	July 2019	–	111	200	311
P	*S. marcescens* LVF3	Myovirus	vB_SmaM-Kodama	July 2019	–	119	198	317
Q	*S. marcescens* LVF3	Myovirus	vB_SmaM-Sureiya	July 2019	–	128	202	330
R	*S. marcescens* LVF3	Myovirus	vB_SmaM-Yubaba	July 2019	–	113	211	324
S	*S. marcescens* LVF3	Siphovirus	vB_SmaS-ChuuTotoro	January 2020	–	81	164	245
T	*S. marcescens* LVF3	Myovirus	vB_SmaM-Kashira	January 2020	–	121	202	323
U	*S. marcescens* LVF3	Siphovirus	vB_SmaS-Kamaji	January 2020	–	78	185	263
V	*S. marcescens* LVF3	Siphovirus	vB_SmaS-ChibiTotoro	January 2020	–	54	125	179
W	*S. marcescens* LVF3	Siphovirus	vB_SmaS-Susuwatari	July 2019	–	61	130	191
X	*S. marcescens* LVF3	Podovirus	vB_SmaP-Kaonashi*	July 2019	–	55	158	213
Y	*S. marcescens* LVF3	Myovirus	vB_SmaM-Otaku	July 2019	–	46	98	144

**Figure 3:**
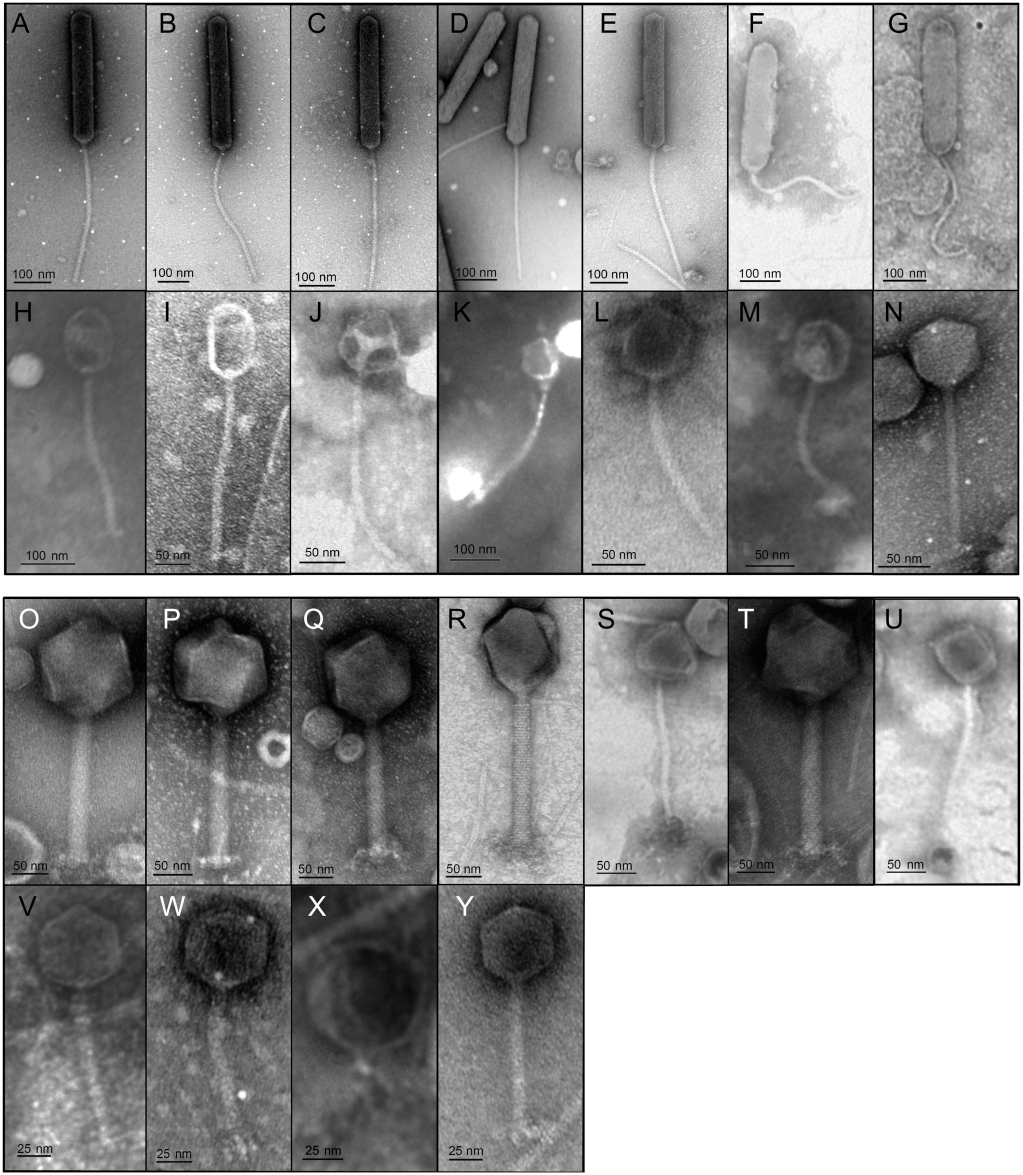
Transmission electron micrographs of all 25 isolates. Bacteriophage preparations were negatively stained with 2% (w/v) aqueous uranyl acetate. Samples were examined in a Joel 1011 transmission electron microscope. The scale bar represent 25 nm **(V–Y)**, 50 nm **(I,J,L–U)** and 100 nm **(A–H,K)**. Table 1 presents the respective physical properties of the shown virions.

Picture ID refers to Figure 3. Tail lengths were measured from the bottom of the neck to the base plate. Bacteriophages were named according to the recommendation described by Adriaenssens and Brister (2017). *Isolation was performed after virome analysis of the dsDNA virome.

Transmission electron microscopy revealed head-tail morphology for all isolates, including 6 myoviruses, 18 siphoviruses, and 1 podovirus with various individual structural features (Figure 3). The isolated *Brevundimonas*-associated phages were all siphoviruses whereas *Serratia* phages revealed three morphotypes (myovirus, siphovirus, and podovirus). Capsid diameter ranged from 45–381 nm and tail length from 98–400 nm (Table 1). Siphoviruses revealed two different types of elongated capsids. The elongation of the shorter type did not exceed twice the diameter of the head, whereas the longer head types frequently exceed three times the head diameter (Figures 3J–Y; Table 1).

### Genome sequencing and characterization

Genomic DNA of each isolate was sequenced and assembled to complete high-quality genomes (Table 2). The genome size of *B. pondensis*-associated phages ranged from 42.3 to 356.9 kb with a G + C content of 49.9 to 65.9% (host G + C content 67.0%). For *Serratia marcescens* LVF3-associated phages, genome sizes ranged from 39.9 to 278.8 kb with a G + C content of 41.0 to 58.6% (host G + C content 59.3%). Phage genome sizes here range from lambda-like phages to jumbo phages (here *Serratia*-associated) or even giant phages (here *Brevundimonas*-associated).

**Table 2:** Genome analyses of all phage isolates associated with *B. pondensis* and *S. marcescens*.

Picture ID	Name	Genome size [bp]	Coverage	G + C % content	CDS	Hypothetical proteins	tRNAs	NCBI accession number
A	vB_BpoS-Papperlapapp	356,874	123.8-fold	65.54	564	475	24	ON529860
B	vB_BpoS-Kabachok	356,285	443.0-fold	65.53	566	486	24	ON529852
C	vB_BpoS-Domovoi	352,705	448.7-fold	65.54	561	473	25	ON529855
D	vB_BpoS-Marchewka	348,421	521.4-fold	65.87	558	473	24	ON529851
E	vB_BpoS-Bambus	348,100	330.6-fold	65.80	550	473	24	ON529853
F	vB_BpoS-Gurke	321,510	98.5-fold	63.08	499	431	29	ON529850
G	vB_BpoS-Kikimora	312,615	175.6-fold	62.85	495	418	29	ON529857
H	vB_BpoS-Poludnitsa	85,956	661.7-fold	61.80	111	88	0	ON529862
I	vB_BpoS-Leszy	85,646	367.0-fold	62.02	113	92	0	ON529856
J	vB_BpoS-StAshley	69,922	66.1-fold	50.07	106	82	0	ON529865
K	vB_BpoS-MaInes	68,997	82.4-fold	49.90	99	75	1	ON529866
L	vB_BpoS-Strzyga	62,088	66.0-fold	59.30	82	60	0	ON529867
M	vB_BpoS-Polewnik	61,859	118.0-fold	59.07	81	59	0	ON529863
N	vB_BpoS-Babayka	42,321	913.2-fold	61.15	53	30	0	ON529868
O	vB_SmaM-Totoro	278,767	314.5-fold	46.27	346	272	1	ON287372
P	vB_SmaM-Kodama	275,052	347.9-fold	46.77	322	255	2	ON287376
Q	vB_SmaM-Sureiya	256,354	316.0-fold	41.04	267	205	4	ON287370
R	vB_SmaM-Yubaba	255,663	66.0-fold	41.05	266	207	4	ON287375
S	vB_SmaS-ChuuTotoro	147,447	47.19-fold	49.19	278	233	19	ON287369
T	vB_SmaM-Kashira	144,511	51.9-fold	50.90	266	221	20	ON287374
U	vB_SmaS-Kamaji	112,334	323.4-fold	44.69	153	100	21	ON287373
V	vB_SmaS-ChibiTotoro	44,971	342.1-fold	57.18	63	37	0	ON287368
W	vB_SmaS-Susuwatari	44,728	246.5-fold	58.62	63	38	0	ON287371
X	vB_SmaP-Kaonashi*	41,649	365.2-fold	53.92	50	20	0	ON287377
Y	vB_SmaM-Otaku	39,857	1,990.0-fold	57.41	62	44	0	ON087563

Picture ID is corresponding to the isolates depicted in Figure 3 and Table 1. Coverage of phages was determined through QualiMap v2.2.2 (Okonechnikov et al., 2016), CDS and hypothetical proteins were determined employing VIBRANT v1.2.1 annotation (Kieft et al., 2020) and manual curation of gene function prediction using InterProScan v5.55–88.0 (Zdobnov and Apweiler, 2001) and Protein BLAST (Altschul et al., 1990). ARAGORN v1.2.41.c (Laslett and Canback, 2004) was used for the detection of tRNAs and tmRNAs (none could be detected). ^*^Isolation was performed after virome analysis of the dsDNA virome.

Annotation of the genomes revealed the presence of phage-specific protein-encoding genes and the presence of tRNA genes frequently. It must be noted that *Brevundimonas*-associated bacteriophages ≥300 kb contained with ≥24 an exceptionally high number of tRNA genes. Similar results were obtained for *Serratia* phages with a genome size of 112 to 148 kb (Table 2).

### Phylogenetic classification of the isolates

**Figure 4:**
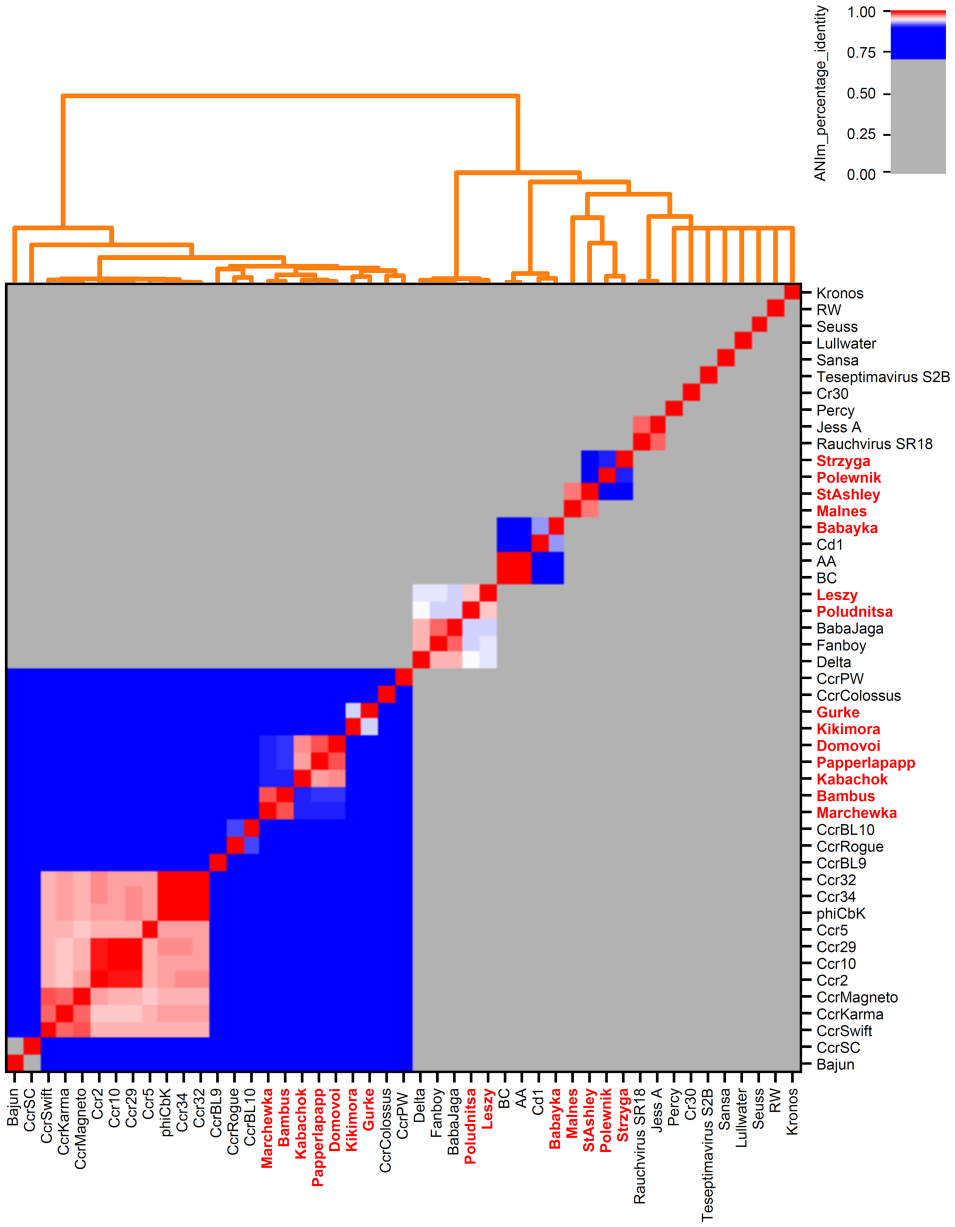
Genome-based phylogenetic analysis of *Caulobacteraceae*-associated bacteriophages. All genomes from NCBI Virus (Brister et al., 2015) and our own isolates (marked in bold red) were examined. Calculations were done with pyani (Pritchard et al., 2016) using ANIm method with default parameters.

We downloaded all publicly available phage genomes associated with the bacterial host genera (Supplementary Tables S2, S3) and used them for a BLASTn-based average nucleotide identity (ANI) analysis. Results revealed five genera which contain two or more species and eight orphan species for phages associated with *Caulobacteracea* (Supplementary Table S2; Figure 4). Only three of our isolates were of the same species (vB_BpoS-Domovoi, vB_BpoS-Papperlapapp, and vB_BpoS-Kabachok). All others were new species or even representatives of new genera (Figure 4). *Serratia-*associated phages were affiliated to 13 genera which contain two or more species and eight orphan species (Supplementary Figure S3; Figure 1). Except vB_SmaM-Kodama, all isolates represented new species, of which at most two were associated with the same genus (Figure 1). These results showed that even with applying the classical isolation technique resulting in isolation of dsDNA phages only, we were able to isolate unknown species and genera. Thus, the plaque technique is certainly not exhausted in its potential and still leads to new discoveries.

### Host-associated viromes

The plates from which we picked plaques and obtained the isolates also served as a starting point to generate host-associated viromes. The plates were washed with medium to collect all present phages, including those unable to form visible plaques and likely being overseen during preceding isolation. Viral derived nucleic acids were used to isolate dsDNA, ssDNA, dsRNA and ssRNA specifically. The amounts recovered were highest for dsDNA (250–400 ng) followed by ssDNA (230–360 ng), ssRNA (60–200 ng) and dsRNA (20–40 ng). The amount of ssRNA compared to the amount of dsDNA seemed relatively high, which might indicate contamination with host RNA.

A total of 16 host-associated metaviromes were studied, consisting of dsDNA, ssDNA, dsRNA and ssRNA metaviromes from two seasons and two host systems. No valuable information could be derived from the RNA viromes associated with *Brevundimonas* and *Serratia* and therefore, the analysis was not considered any further (Supplementary Table S5). In contrast, the amount of dsDNA and ssDNA was high (Table 3). The assembled dsDNA virome of *B. pondensis* LVF1 comprised 13 (winter season) and 334 (summer season) contigs. The winter and summer season ssDNA viromes contained 16 and 134 contigs, respectively.

**Table 3:** dsDNA and ssDNA viromes associated with *Brevundimonas pondensis* and *Serratia marcescens*.

Virome	Number of reads after host removal	Average length of sequences (N50) [bp]	Number of contigs	Unique contigs	Unique and circular contigs	Unique and circular contigs phage-associated	Unique and non-circular contigs phage-associated	Sample-specific contigs
LVF1_p1_dsDNA	2,258,351	238,301	13	0	0	0	0	0
LVF1_p1_ssDNA	833,717	29,017	16	0	0	0	0	0
LVF1_p2_dsDNA	1,780,267	8,002	334	12	0	0	5	7
LVF1_p2_ssDNA	733,624	5,508	134	18	0	0	14	4
LVF3_p1_dsDNA	2,916,604	10,104	329	183	1	1	180	2
LVF3_p1_ssDNA	2,372,672	5,508	1	0	0	0	0	0
LVF3_p2_dsDNA	2,913,723	39,857	26	13	0	0	13	0
LVF3_p2_ssDNA	2,391,113	39,857	1	0	0	0	0	0

LVF1_p1 stands for the virome associated with *Brevundimonas pondensis* LVF1 from January 2019, while LVF1_p2 is from July 2019. LVF3_p1 stands for the virome associated with *Serratia marcescens* LVF3 from July 2019, while LVF3_p2 is from January 2020. An overview of number of reads after host removal, the average length of sequences (N50) [bp], number of contigs (consensus region of DNA after Unicycler assembly and further decontamination), unique, and unique and circular contigs (all contigs which do not map with isolates and might be circular), unique and circular associated with our host systems (contigs which show sequence-similarity with other *Brevundimonas*-, *Caulobacter*- and *Serratia*-associated phages and are unique), unique and non-circular associated with our host systems contigs (same as before but with non-circular contigs), and sample-specific contigs (closest Blastp and VIBRANT results reveal non-phage associated contigs) are listed.

The dsDNA metavirome associated with *S. marcescens* LVF3 led to 329 contigs for the summer season and only 26 for the winter season. The ssDNA viromes of both seasons exhibited a total of one circular contig with the same size of 39,857 bp, implying one phage associated with *Serratia* (dsDNA phage vB_SmaM-Otaku).

In summary, the data (Table 3) indicated that phage diversity is influenced by seasonal changes. Our results suggested that the phage diversity was higher in summer than in winter and that dsDNA phages were the dominant group associated to the hosts in the environment.

### Virome entities not covered by *Brevundimonas* and *Serratia-*associated phage isolates

To investigate which proportion of the viromes matched our isolates (Table 3), we compared the contigs from the host-associated viromes to the genomes of the viral isolates at sequence level (Supplementary Tables S6–S13).

For the winter season of the LVF1-associated dsDNA virome, all contigs matched our isolates, meaning the isolation was holistic, and we did not miss any individual phage. In contrast, 322 of 334 contigs of the summer season revealed similarity to our phages, and remaining 12 contigs were unique. Six of these contigs were determined as phage-associated using VIBRANT analysis (Kieft et al., 2020). The other six contigs were sample-specific (sequences only present in the sample without being phage-associated). Contigs not associated with our isolates indicated a diversity of close-related phages. Investigation of the potential protein-encoding genes predicted from the unique contigs revealed similarities to DNA primases, phage terminases (large subunit), minor tail proteins, tail tip proteins, tail assembly proteins and putative baseplate hub proteins, DNA ligases and DNA polymerases (Supplementary Data File S1). Thus, these contigs were also phage-derived.

For the summer season of the *S. marcescens* LVF3-associated dsDNA virome, 183 of the 329 non-circular contigs were not associated with our isolates. One hundred eighty of these contigs were phage-associated, and one of the circular contigs implied a complete phage genome. The remaining two contigs were sample-specific. Noteworthy, some of the phage-associated contigs revealed sequence similarity to known phages associated with *Cronobacter, Erwinia, Escherichia, Salmonella*, and *Pseudomonas* (Supplementary Data File S2), implying a broad host range. In contrast, 13 of the 26 contigs of the winter season revealed similarity to our phages, and 13 remained unique. We observed protein-encoding genes similar to tail tube proteins, putative virion structural proteins, DNA primases, ATP-dependent helicase, viral DNA polymerases, putative tail sheath protein and DNA ligase. The 13 unique contigs of the winter virome encoded putative virion structural proteins, DNA polymerase, major capsid protein, helicase, and putative tail sheath protein (Supplementary Data File S1).

To analyze the isolated viral fraction, we investigated the proportion of the sequences associated with our isolates (Figure 2). Phage isolates associated with *B. pondensis* LVF1 comprised 67.4% of the dsDNA reads of the summer season and 96.1% of the winter season, whereas dsDNA reads of *S. marcescens* LVF3 comprised 76.4 and 94.0% of the summer and winter season, respectively. Both results revealed the main fraction of the dsDNA virome was successfully addressed by the overlay plaque assay. To analyze the presence of known but not isolated phages, we also mapped the reads on the genomes of related phages obtained from GenBank (accessed on January 20, 2022; Supplementary Tables S14–S17). The phage diversity seemed to be highest for *B. pondensis* LVF1 and *S. marcescens* LVF3 during the summer season. The dominant phage for LVF1 was vB_BpoS-Domovoi (24.6%), while during the winter season this phage was barely detectable (1.0%). In contrast, vB_BpoS-Bambus was in summer almost absent (2.1%), while in winter dominant (62.9%). Phage diversity in the summer season of the *S. marcescens* virome was also high. The three dominant phages in the summer season were vB_SmaM-Otaku (18.9%), vB_SmaM-Sureiya (16.6%) and vB_SmaM-Yubaba (18.6%). In the winter season, vB_SmaM-Otaku (81.6%) was the prominent phage isolate, followed by vB_SmaM-Kashira (8.7%). The dsDNA viromes associated with LVF3 contained known *Serratia* phages, which were not isolated. These comprised 2050HW (2%), BUCT660 (2%), Moabite (1.6%), vB_SmaM_Hyamo (1.6%), vB_SmaM_Yaphecito (2.2%) and Tsm2 (2.0%).

We conclude from the dsDNA virome results that we can efficiently isolate dsDNA phages using the plaque overlay method. However, depending on the sample, a considerable diversity remains unavailable, but we isolated the majority of the dominant phages.

### A phage from the dsDNA virome

Virome contigs not belonging to the isolates could be assembled into a circular unit. Thus, it likely represents a complete phage genome, which provides a chance to isolate the respective phage from the remaining sample. In this way, phage vB_SmaP-Kaonashi (41,649 bp) (Figure 3) was identified through a specific PCR screening applied on various subsequently generated plaques and successfully isolated (Tables 1, 2). This example highlights the potential of accompanying host-associated metavirome analysis.

### A broad host dsDNA phage isolate

One of the frequent circular contigs associated with both host systems – *B. pondensis* and *S. marcescens* was phage vB_SmaM-Otaku (39,857 bp). We observed its presence in the *B. pondensis* LVF1 dsDNA virome of the summer season. We were able to isolate and characterize the phage genomically and morphologically. Through several reinfections *via* Overlay Plaque Assay of *S. marcescens* with vB_SmaM-Otaku, we received a pure phage isolate. A PCR screening confirmed its presence in the *B. pondensis*-associated metaviral sample as well. An infection of *B. pondensis* with the purified vB_SmaM-Otaku confirmed the ability of a broad-host infection as we could confirm its presence by revealing unequivocal plaques on an overlay assay (data not shown) and through PCR screening. The number of plaques produced by phage Otaku was in *B.* pondensis (~20) much lower than in *S. marcescens* (~150). Further, through several reinfections *via* Overlay Plaque Assay of *B. pondensis* with phage Otaku infecting *Brevundimonas*, a pure phage isolate was generated. This pure Otaku phage isolate was used for the infection of *S. marcescens*. The number of plaques produced by re-introducing phage Otaku into *Serratia* showed a similar amount (~135), The PCR screening confirmed the presence of this phage. Therefore, we concluded that vB_SmaM-Otaku is not a contamination, it is a phage with a broad host range.

### ssDNA viromes

Since we have not been able to isolate phages other than dsDNA phages, viromes based on a distinct nucleic acid were of particular interest. For the 17 ssDNA *B. pondensis* LVF1 virome-associated contigs from the winter season, all contigs did align to known dsDNA phages vB_BpoS-MaInes and vB_BpoS-StAshley. For the summer season, 115 of 134 contigs showed sequence similarity to known dsDNA phages. Of the remaining 18 unique contigs, 14 were predicted as phage-associated. Some revealed sequence similarity to *Acinetobacter-* (contig 1) and *Bacillus*-associated (contigs 58 and 71) phages, but also to a *Siphoviridae* sp. isolate ctfaf4 (contig 74) and unknown bacteriophage sp. isolate ctu5M1 (contig 106). Some of the phage-associated contigs had no Blastn hits, although VIBRANT predicted some of them as phage-associated (contigs 119, 162, and 289) containing genes coding for typical phage proteins such as portal protein, tail sheath protein, DNA ligase and terminase. Seven of the unique contigs (contig 148, 167, 494, 634, 638, 700, and 707) were sample-specific. Also, the prediction of functional protein domains of the annotated genes resulted in the closest hit with, e.g., 30% sequence identity with a DNA gyrase subunit B from *Bacillus* phage SP-15. The highest amino acid sequence identity (50%) of a protein sequence derived from the contig was to a hypothetical protein from vB_BpoS-Kikimora.

**Figure 5:**
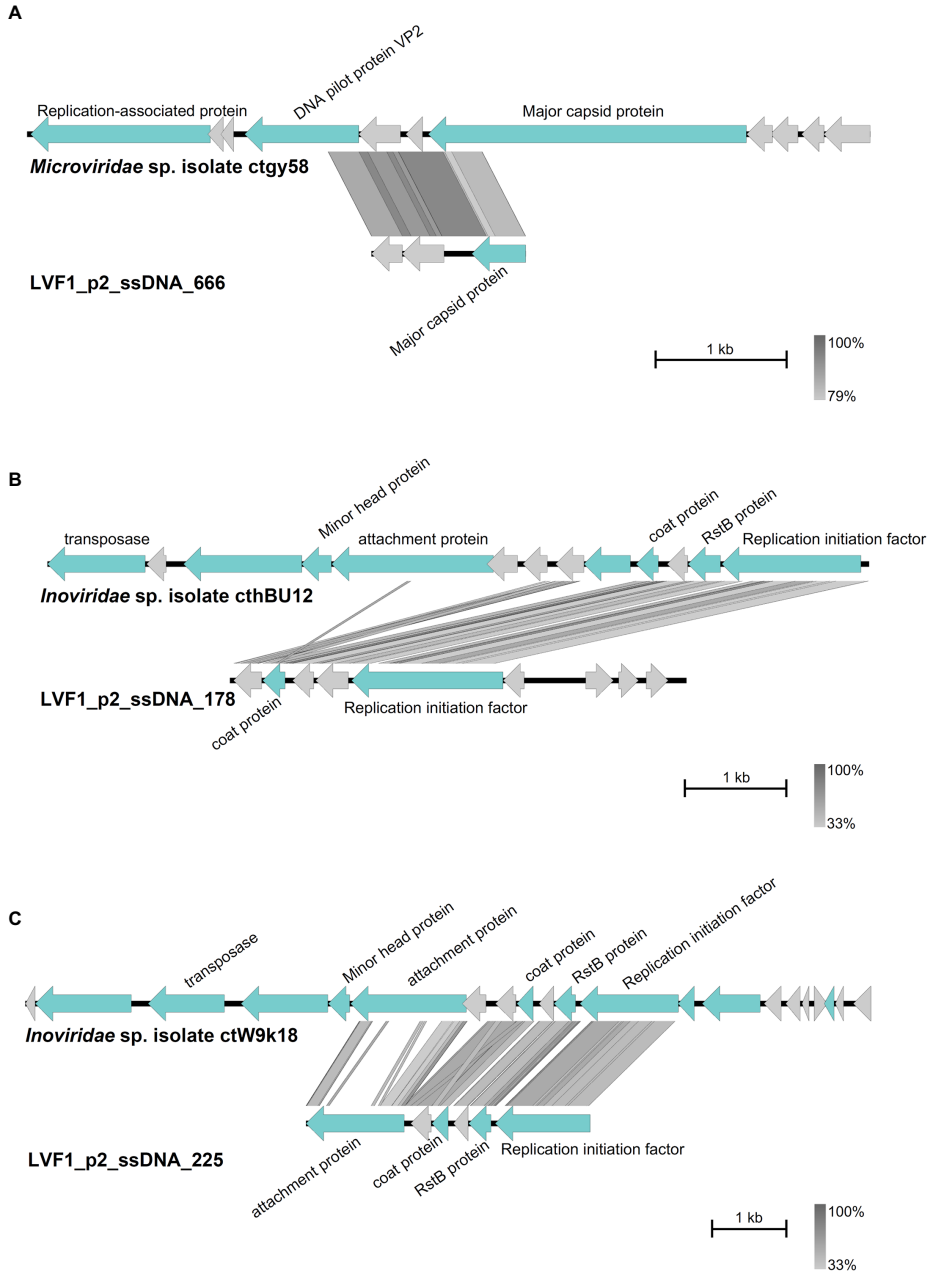
Comparison of the contigs with best Blastn matches. Arrow indicates gene direction. Phage-specific gene products are shown in light blue with corresponding labeling, hypothetical proteins in light grey. Comparison of **(A)** contig 666 with *Microviridae* sp. isolate ctgy58, **(B)** contig 178 with *Inoviridae* sp. isolate cthBU12 and **(C)** contig 225 with *Inoviridae* sp. isolate ctW9k18. Plot was created with Easyfig (Sullivan et al., 2011).

Noteworthy, we could identify high sequence similarity of contig 666 to *Microviridae* sp. isolate ctwNz7 (Figure 5A) and of contigs 178 and 225 to *Inoviridae* sp. isolate ctoJk8/ctDT74 (Figures 5B,C). These two phage families use ssDNA as genomic material. Predicted proteins derived from the ssDNA contigs (*Inoviridae* and *Microviridae* hits) were similar to coat proteins, attachment proteins, RstB proteins and replication initiation proteins. Thus, although our virome ssDNA preparation was imperfect and contained a dsDNA fragments, we were able to detect the presence of ssDNA phages.

## Discussion

### Host system selection

The bacterial strains *Brevundimonas* and *Serratia* were associated with diverse DNA and RNA viruses. Fukuda et al. (1976) successfully isolated dsDNA giant phage (φCp34) associated with *Caulobacter crescentus*. In addition, RNA phages were isolated by the group around the same time (Miyakawa et al., 1976). The ssDNA phage φX174 (Sanger et al., 1977), dsDNA phage T7 (Demerec and Fano, 1945) and ssRNA phage MS2 (Davis et al., 1961) were associated with the genus *Escherichia*. Given the taxonomic proximity of *Serratia* to *Escherichia* and *Brevundimonas* to *Caulobacter*, we anticipated high viral diversity associated with *B. pondensis* LVF1 and *S. marcescens* LVF3. The preference for our host over established host systems was to ensure that, even when phage diversity was low, the isolated phages would likely be unique and contribute to viral diversity exploitation.

From our 25 isolates, five of the *Caulobacteraceae*-associated phages and seven of the *Serratia*-associated phages belong to new genera, underlining that there is still much to discover even with classical methods by employing new prokaryotic host systems. These comprised *Brevundimonas*-associated phages vB_BpoS-Strzyga, vB_BpoS-Polewnik, vB_BpoS-StAshley, vB_BpoS-MaInes, and vB_BpoS-Babayka. All five belonged to the same genus. In the case of *Serratia*, vB_SmaP-Kaonashi, vB_SmaS-ChuuTotoro, vB_SmaM-Kashira, vB_SmaS-ChibiTotoro, vB_SmaS-Susuwatari, vB_SmaM-Yubaba, and vB_SmaM-Sureiya were distributed over four new phage genera. In comparison, no new viral genus of *Escherichia* phages has been described for decades to our knowledge. We were particularly surprised by the high number of jumbo and giant phages among the new isolates. Although jumbo phages like 201φ2-1 (Thomas et al., 2008) and CcrColossus (Gill et al., 2012) for the host systems *Pseudomonas* and *Caulobacter* as well as giant phages PA5oct and pEa_SNUABM_44 (Drulis-Kawa et al., 2014; Kim et al., 2020) for *Pseudomonas* and *Erwinia* host systems have already been described, these phages have rarely been observed, especially in model host systems despite various attempts (Schilling et al., 2018a,b; Nordmann et al., 2019; Furrer et al., 2020). For example, only two species of jumbo phages were known to be associated with the model organism *Bacillus subtilis*, including the group of PBS1-like phages represented by the isolates PBS1 (Eiserling, 1967) and AR9 (Lavysh et al., 2016), and SP10. The latter has never been reisolated for more than half a century.

The reasons for the success in employing our host systems for the isolation of large phages are unknown. Nevertheless, it is evident that the slow-growing *B. pondensis* LVF1 (Friedrich et al., 2021b) led to the isolation of jumbo and giant phages rather than the faster-growing *S. marcescens* LVF3 (Friedrich et al., 2021a). The growth characteristics of LVF3 were very similar to that of the *Escherichia coli* model, and the isolated phage vB_SmaS-ChibiTotoro and vB_SmaS-Susuwatari also strongly resembled the known *Escherichia* phage Lambda morphologically and genomically (King et al., 2012c). Thus, we assume that host systems with slower growth rate give the larger phages more time to reproduce with their prolonged vegetative period and lead to a visible plaque on agar plates. We are unaware that phage isolation was tried on minimal media with established model host systems. Bacteria growth is at a much lower rate on minimal media, which prolongs the vegetative phases. Thus, it would be interesting to explore such conditions for the isolation of jumbo and giant phages of model host systems such as *E. coli*, *B. subtilis* and our *S. marcescens*.

We demonstrated that the overlay assay was able to grasp most of the viral dsDNA diversity. We could isolate most of the bacteriophages associated with both host strains, as confirmed by the host-associated metavirome data. Nevertheless, differences between the host systems were encountered and a seasonal impact was indicated. Further, we showed that *S. marcescens*-associated virome contained many phage-associated contigs, which are not part of the known phages infecting the *Yersiniaceae* family. These were *Erwinia*-, *Salmonella*- or *Cronobacter*-associated phages. They might not efficiently infect *S. marcescens*, but *S. marcescens*-associated phages show a broad host spectrum, i.e., the *Serratia* phage vB_SmaM-Otaku, which is able to infect *B. pondensis* (lysis was observed). Accordingly, *Serratia* phages are often able to infect related genera (Prinsloo and Coetzee, 1964; Prinsloo, 1966; Evans et al., 2010).

### Viromes reveal a plethora of undetected host-associated phages

The dsDNA virome analysis showed that isolation with a classical plaque assay is very efficient and allows the recovery of the main present viral diversity. However, the complementary isolation of phages not initially detected demonstrated the value of accompanying host-associated metavirome analysis. For example, phage vB_SmaP-Kaonashi could only be isolated after identification in the corresponding virome dataset. Noteworthy, an important outcome of virome analysis was the identification of an isolate with a broad host range. The alignment of reads against a foreign host system led to the identification of vB_SmaM-Otaku, which experimentally proved to infect both *B. pondensis* LVF1 and *S. marcescens* LVF3 successfully.

The low concentration of ssDNA and the with dsDNA contaminated ssDNA virome sequences imply that only very few ssDNA phages were present in our samples; thereby explaining the lack of isolates. However, this would be too simplistic. Note that classical isolation methods fundamentally discriminate against this group of phages. *Inoviridae* infections are not lethal and do not necessarily lead to a visible plaque, which is necessary to identify and isolate a phage. In addition, we were able to detect *Microviridae*-like phages using TEM of the host-associated virome sample from the summer season (data not shown). These were round and non-tailed with an icosahedral symmetry and a diameter of roughly 30 nm. Further, we were also able to detect filamentous structures (*Inoviridae*-like) in the metaviral sample. Nevertheless, ssDNA virome analysis has demonstrated that at least the *B. pondensis* LVF1 system is associated with *Microviridae* and *Inoviridae*. Thus, new or specifically optimized experimental approaches will be required to access these phages.

The situation is similar with RNA phages, and we have not succeeded in obtaining RNA phage isolates or virome-derived RNA phage sequences with both host systems. The nucleic acid amount of the dsRNA and ssRNA virome was low, except for the *B. pondensis* ssRNA virome from summer season. Further analysis confirmed contamination with ribosomal host RNA. An optimization of the methodology would be needed by getting rid of the host RNA and DNA. In addition, compared to DNA phages, RNA phages are much smaller regarding their genomic sizes [ssRNA phages 3.5–4.3 kb (King et al., 2012b) and dsRNA phages 12.7–15.0 kb (King et al., 2012a)]. Therefore, we would suspect a higher DNA to RNA base ratio. Further, virome samples typically represent low-abundance viruses better than intracellular viral genomes such as non-replicating proviruses and virocells (Howard-Varona et al., 2020).

Again, we conclude that there is a need for novel approaches to access this realm of viral diversity rather than RNA phages being not associated with our hosts. Nevertheless, in a different study with *B. goettingensis*, we were able to discover the genome of an ssRNA phage of the *Leviviridae* family with the same nucleic acid isolation procedure (Friedrich et al., unpublished results).

## Conclusion

We showed that the classical phage isolation methodology still bears a great potential to detect organismic and genetic phage diversity as we were able to isolate 14 *Brevundimonas*- and 11 *Serratia*-associated phages. While the morphological and genomic diversity of *Serratia*-associated phages appears to be greater than that of *Brevundimonas*, the *Brevundimonas*-associated virome revealed other phage genome types, e.g., ssDNA phages. Nevertheless, the classical method has its limitations such as only the isolation of particle-protected and plaque-forming phages. The range of host-associated phages can be expanded by complementation with sequencing-based metavirome analysis approaches, but the limitations cannot be entirely solved by employing these strategies.

## Data availability statement

The datasets presented in this study can be found in online repositories. The names of the repository/repositories and accession number(s) can be found in the article/Supplementary material.

## Author contributions

IF, RH, and RD conceptualized and designed the study. IF, HN, AK, BB, FT, SH, AP, and MB performed the experiments. IF, HN, AK, MH, and DS performed the visualization of results. IF and RH wrote the first draft of the manuscript. RD revised the manuscript. All authors interpreted the results and reviewed the final version of the manuscript.

## Funding

We acknowledge support by the Open Access Publication Funds of the University of Göttingen, which had no role in study design, data collection, and interpretation, or the decision to submit the work for publication.

## Conflict of interest

The authors declare that the research was conducted in the absence of any commercial or financial relationships that could be construed as a potential conflict of interest.

## Publisher’s note

All claims expressed in this article are solely those of the authors and do not necessarily represent those of their affiliated organizations, or those of the publisher, the editors and the reviewers. Any product that may be evaluated in this article, or claim that may be made by its manufacturer, is not guaranteed or endorsed by the publisher.
