# Supplementary material for: Brevundimonas and Serratia as host systems for assessing associated environmental viromes and phage diversity by complementary approaches
Source: Front Microbiol. 2023 Mar 21;14:1095850. doi: 10.3389/fmicb.2023.1095850 (PMC10070969; doi:10.3389/fmicb.2023.1095850)
Supplement: Supplementary file 4 [file Image_1.pdf]

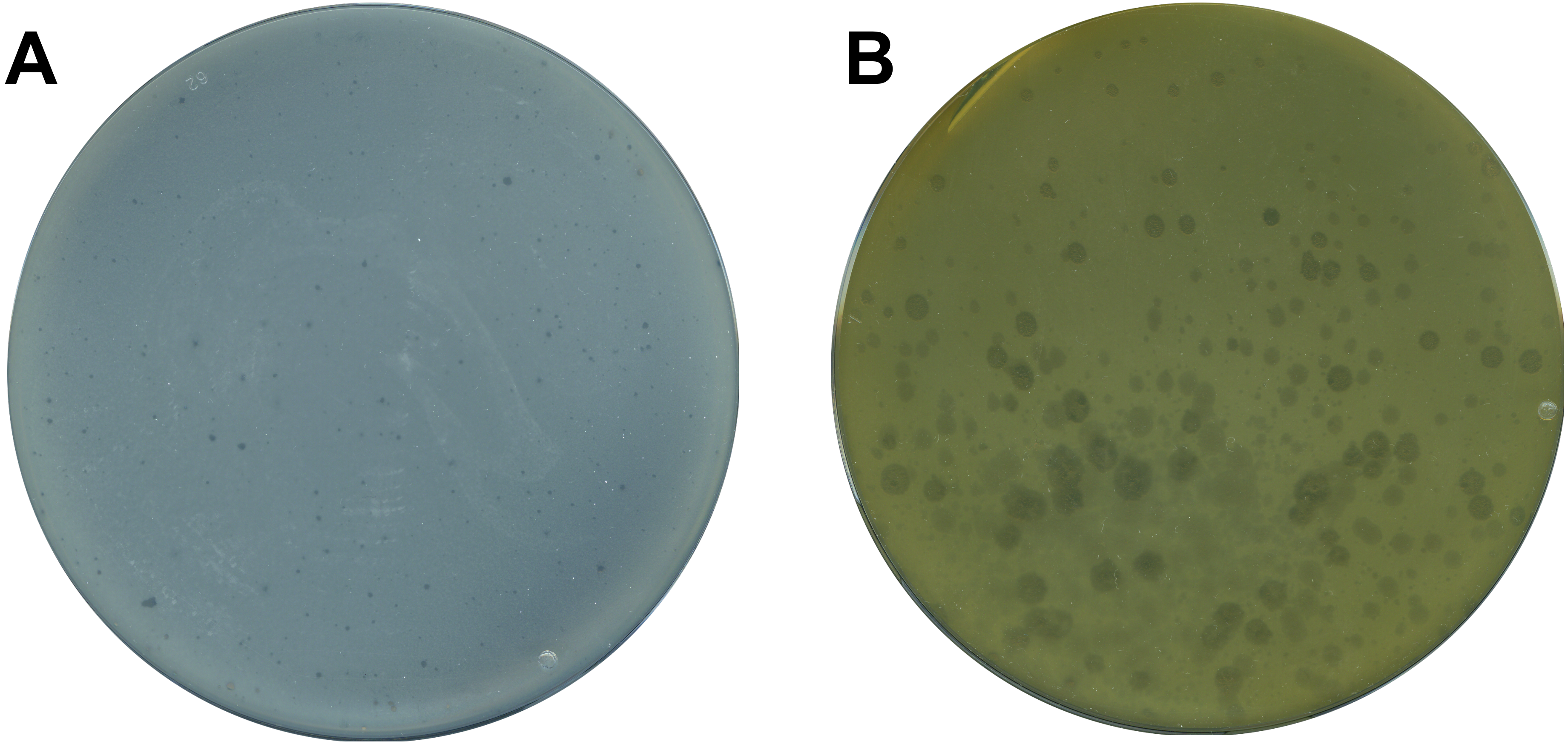

Supplement Figure S1. *Brevundimonas pondensis* LVF1 and *Serratia marcescens* LVF3 challenged with sewage phage suspension. Different plaque morphologies can be observed.
